# Efficacy and Safety of Different Formulations of Calcipotriol/Betamethasone Dipropionate in Psoriasis: Gel, Foam, and Ointment

**DOI:** 10.3390/jcm10235589

**Published:** 2021-11-28

**Authors:** Lidia Rudnicka, Małgorzata Olszewska, Mohamad Goldust, Anna Waśkiel-Burnat, Olga Warszawik-Hendzel, Przemysław Dorożyński, Jadwiga Turło, Adriana Rakowska

**Affiliations:** 1 Department of Dermatology, Medical University of Warsaw, 02-008 Warsaw, Poland; malgorzata.olszewska@wum.edu.pl (M.O.); anna.waskiel@wum.edu.pl (A.W.-B.); olga.warszawik-hendzel@wum.edu.pl (O.W.-H.); adriana.rakowska@wum.edu.pl (A.R.); 2 Department of Dermatology, University Medical Center of the Johannes Gutenberg University, 55122 Mainz, Germany; drmgjgoldust@gmail.com; 3 Department of Drug Technology and Pharmaceutical Biotechnology, Medical University of Warsaw, 02-097 Warsaw, Poland; pdorozynski@wum.edu.pl (P.D.); jadwiga.turlo@wum.edu.pl (J.T.)

**Keywords:** calcipotriol, betamethasone dipropionate, long-term treatment, nail psoriasis, proactive treatment, psoriasis, topical therapy, treatment, scalp psoriasis, vitamin D3 derivatives

## Abstract

Preparations containing calcipotriol combined with betamethasone dipropionate (in the forms of ointment, gel, and foam) are available for the topical treatment of psoriasis. This review summarizes the differences in the efficacy and safety of these formulations, as well as the preferences of patients with various forms of psoriasis (plaque, scalp, and nail psoriasis). It has been documented that foams provide higher bioavailability, resulting in increased efficacy in plaque psoriasis compared to ointments and gels. Gels or foams are preferred by patients for their different practical qualities (e.g., gels for “easy application”, and foams for “immediate relief”). The available data indicate that ointments may be the most effective formulation in nail psoriasis, and gels are preferred by patients with scalp psoriasis because of their cosmetic features. Treatment with a foam formulation is associated with a lower number of medical appointments compared to treatment with an ointment and with a lower probability of developing indications for systemic treatment. The safety profiles of foams, ointments, and gels are comparable, with the most common adverse effect being pruritus at the application site (in 5.8% of the patients). A long-term proactive maintenance therapy markedly reduces the number of relapses and is likely to close the gap between topical and systemic treatment in psoriasis.

## 1. Introduction

Preparations containing calcipotriol combined with betamethasone (in the form of betamethasone dipropionate) are available for the topical treatment of mild psoriasis [1,2]. The topical formulations of calcipotriol with betamethasone available in most countries are ointments, gels, and foams [3,4,5,6]. The specific properties of the these preparations may not be comprehensible to every clinical practitioner. 

The aim of this article is to review the similarities and differences between these three formulations. In this article, we use the terms “foam” and “foam containing calcipotriol with betamethasone” interchangeably as equivalent to “foam containing calcipotriol with betamethasone dipropionate”, the terms “ointment” and “ointment containing calcipotriol with betamethasone” as equivalent to “ointment containing calcipotriol with betamethasone dipropionate”, and the terms “gel” or “gel containing calcipotriol with betamethasone” as equivalent to “gel containing calcipotriol with betamethasone dipropionate”. All data refer to the approved calcitriol/betamethasone dipropionate concentration of 0.005%/0.064%. As of 2021, the concentration of active ingredients is identical in all formulations across all countries. The literature search for this article was performed until 31 January 2021 in PubMed and Scopus, using the search terms “calcipotriol” and “betamethasone” with “ointment” or “gel” or “foam”. All search results were analyzed in detail.

## 2. Pharmacodynamics of Calcipotriol/Betamethasone Dipropionate

Calcipotriol, a synthetic vitamin D3 analogue, has a similar mode of action to calcitriol—changing the expression of genes responsive to vitamin D. It binds to the retinoid X receptor and influences cell differentiation and growth regulation, immune functions, and the balance of calcium and phosphorus in the body [7]. It also has a reductive effect on the hyperproliferation of keratinocytes, normalizes their differentiation, and reduces the pro-inflammatory cytokine level, which induces anti-inflammatory and immunomodulatory effects [7,8]. 

Betamethasone dipropionate belongs to the group of synthetic fluorinated glucocorticoids that exhibit anti-inflammatory and immunosuppressive effects by binding to glucocorticoid cytosolic receptors and then translocating to the nucleus where they regulate the transcription of numerous genes responsible for the immune response. It limits inflammatory infiltration, erythema, and edema, inhibits cell hyperproliferation, and improves the differentiation of keratinocytes in psoriasis [8,9]. 

Pharmacodynamic studies showed the anti-inflammatory and immunoregulatory synergy of the combination of calcipotriol and betamethasone dipropionate with respect to the effects of these active substances administered individually [10]. The effectiveness of calcipotriol/betamethasone dipropionate mixtures is related to a synergy of action of the two substances. Calcipotriol affects keratinocyte differentiation, while betamethasone influences inflammatory processes and minimizes skin irritation (e.g., pruritus) after calcipotriol application [11]. 

The mechanism of calcipotriol/betamethasoneantipsoriatic activity has remained only partially known for a long time. However, in the last decade, a number of publications have started to discuss the immune background of psoriasis and the influence of T cells, B cells, dendric cells, as well as cytokines in its pathogenesis [12,13,14,15]. A novel approach to the investigation of the mechanism of action of therapeutics applied in psoriasis treatment has been proposed. Recently, Satake et al. [16] have investigated the synergistic effects of drug substances in combination therapy with Cal/BS for dermatitis-like psoriasis. They investigated the basic immune mechanisms in a mouse model of imiquimod-induced psoriasis. Cal/BS combination appeared effective in inhibiting the effects induced by imiquimod in comparison with a monotherapy with calcipotriol or betamethasone. The authors emphasized that Cal/BS synergistically induced CD8^+^ regulatory T cells and improved the balance between CD8^+^ or CD4^+^ regulatory T cells and pro-inflammatory CCR6^+^ γδ T17 lymphocytes in the lymph nodes. The data indicated that the synergistic antipsoriatic effect of Cal/BS was based on a reduction of the imbalance between regulatory CD8^+^ or CD4^+^ T cells and pro-inflammatory CCR6^+^ γδ T17 cells.

Calcipotriol is stable in alkaline solutions with pH above 8, whereas betamethasone dipropionate requires an acidic environment with pH between 4 and 6. Therefore, the presence of both substances in an aqueous environment leads to interactions and to their decomposition [8]. For this reason, the treatment of psoriasis with calcipotriol and betamethasone dipropionate was initially carried out by applying them separately twice a day or sequentially [10]. The development of a formulation type of fixed dose combinations created the possibility of the simultaneous application of calcipotriol and betamethasone dipropionate, increasing their effectiveness and convenience of use, as well as patients’ compliance [17]. The treatment is safe, systemic exposure after topical administration of calcipotriol and betamethasone dipropionate is low, and the absorption of the substances after application to healthy skin does not exceed 1%. In patients with psoriasis, the blood levels of the drugs were below the quantification level after 4 to 8 weeks of treatment [7].

## 3. Supersaturated Foam Formulation of Calcipotriol/Betamethasone 

Dermal and transdermal drug delivery is a continuous challenge for pharmaceutical technology. A number of various strategies of drug delivery across the skin barrier have been tested through the decades. A rich set of methods has also been proposed and tested for psoriasis treatment, e.g., laser-assisted drug delivery, foam formulations, nanoparticles, ethosomes, niomes [18]. Among these methods, the application of supersaturated solutions is widely accepted for the treatment with calcipotriol/betamethasone formulations [19]. The penetration of the skin by active substances after topical application is directly proportional to their concentration. Low solubility in the vehicle is a limitation for the majority of active substances in topical preparations. The chemical potential of a substance may be “artificially” increased above its solubility by using supersaturated solutions, which gives the opportunity to improve its delivery. Supersaturated solutions are thermodynamically unstable but they may be temporarily stabilized during treatment.

**Figure 1:**
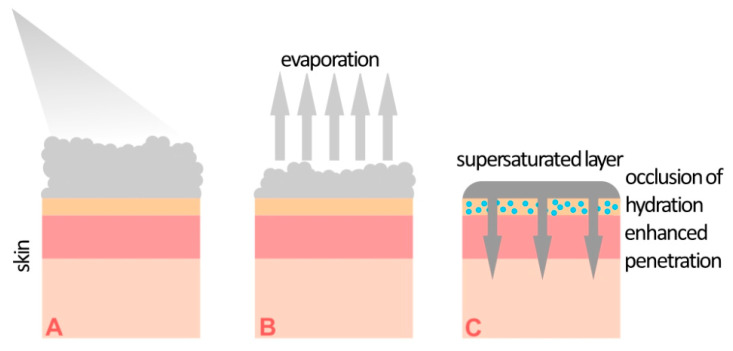
Illustration of the formation of a supersaturated layer on the skin after the administration of a calcipotriol/betamethasone foam. (**A**) Foam application, (**B**) solvent evaporation, (**C**) formation of a supersaturated layer.

Supersaturation, involving the increased concentration of a substance above its vehicle solubility threshold, was introduced for foams containing calcipotriol and betamethasone [19,20]. A supersaturated solution is formed on the skin surface ex tempore after the application of the preparation through immediate propellant evaporation. According to the information in the Summary of Product Characteristics (SmPC) of commercial foam formulations containing calcipotriol/betamethasone, the following main excipients are present: white petroleum, polyoxypropylene stearyl ether, liquid paraffin, butane, and dimethyl ether. According to their role in foam formulations, these substances may be divided into two groups, i.e., lipid anhydrous bases for calcipotriol/betamethasone and volatile solvents, which also act as propellants. The non-aqueous environment protects the active substances from decomposition due to their pH sensitivity. The processes occurring after foam application are shown in Figure 1. After application, butane and dimethyl ether, whose boiling points are below 0 °C, quickly evaporate, leaving a supersaturated solution of calcipotriol/betamethasone in a lipid basis on the surface of the skin. A study presented by Lind et al. [19] showed that the propellant concentration within the foam was reduced to below 2% within 30 s. Microscopy, Raman imaging, and X-ray powder diffraction (XRPD) studies confirmed that the active substances do not recrystallize in foam formulations for at least 18 h, and probably much longer. In contrast, crystals were observed immediately after the application of a standard ointment formulation. Research on the penetration of calcipotriol and betamethasone dipropionate through pig ear skin confirmed a statistically significant increase in active substance concentrations in comparison with the concentrations reached when using an ointment. From a practical point of view, the occlusive properties of the supersaturated layer are very important. They increase the hydration of the stratum corneum by inhibiting water evaporation, which improves skin permeability. 

Both in vitro and in vivo research showed the superiority in active substances’ speed of penetration and concentration reached when using foams compared to gels or ointments [19].

The outcomes obtained with the use of foams support the view of an increasing number of dermatologists that foams leading to supersaturation of calcipotriol and betamethasone will change dermatology and clinical practice as regards the treatment of psoriasis [21,22]. 

## 4. Comparison of Foam and Ointment 

A double-blind multicenter phase II study [23] compared the effectiveness and safety of two preparations containing calcipotriol and betamethasone dipropionate—a foam and an ointment. The study included a total of 376 patients. The primary endpoint to evaluate the formulations’ effectiveness was the percentage of patients whose skin lesions regressed or almost completely regressed as confirmed by Physicians Global Assessment (PGA) analysis after 4 weeks. The number of patients in whom therapeutic success was achieved was significantly higher in the group who had used the foam formulation compared to the group who had used the ointment (54.6% and 43.0%, respectively, *p* = 0.025). Moreover, assessment with the mPASI method (modification of the Psoriasis Area and Severity Index, which excludes the hairy scalp on which no foam/ointment was applied) demonstrated a statistically significant advantage of foam over ointment at both assessed time points (1 week and 4 weeks). The authors concluded that the effectiveness of the foam formulation was markedly higher than that of the ointment, with a comparable safety profile.

The standard vasoconstriction test for the evaluation of glucocorticoid effects was used to compare the activities of a foam containing calcipotriol and betamethasone and an ointment containing betamethasone (no calcipotriol). The degree of vasoconstriction obtained with the foam was (median) 2.00 points, while that achieved with the ointment containing betamethasone but without calcipotriol was 1.75 points, with the difference being statistically insignificant (*p* = 0.30) [24].

An analysis of the cost effectiveness of foams and ointments containing calcipotriol and betamethasone was conducted in Sweden [25]. A relatively complex organizational regimen involved the application of a foam or an ointment prior to systemic treatment. A significantly higher effectiveness of the foam was observed in comparison with the ointment. The use of foam was associated with a lower number of medical consultations and a lower percentage of patients for whom systemic treatment was necessary. 

## 5. Comparison of Foam and Gel 

A 12-week PSO-ABLE phase III study [26] was conducted to compare the therapeutic effectiveness and safety of a foam containing calcipotriol and betamethasone and a gel containing the same amount of active substances. The study included 463 patients. The average baseline BSA was 7.1 ± 5.7 in the group of patients using the foam and 7.0 ± 5.5 in the group who used the gel. The primary endpoint of effectiveness assessment was the percentage of patients in whom therapeutic success was achieved. Therapeutic success was defined according to the PGA scale (0–4) as “no lesions” in case of patients with mild lesions at baseline and “no lesions” or “almost no lesions” in patients with moderate or severe psoriasis at baseline. On the basis of the above definition, the effectiveness of the foam was characterized as markedly higher compared to that of the gel. The percentages of patients in whom therapeutic success was achieved (“no” or “almost no” psoriatic lesions) were 38.3% and 19.6% after 4 weeks for the foam and gel groups, respectively, while after 8 weeks, the respective percentages were 44.5% and 22.5%. The study also showed that, after 4 weeks, therapeutic success was achieved in a significantly higher percentage of patients using the foam, whereas it required 8 weeks in patients using the gel. A similar difference was observed when analyzing the effectiveness with mPASI. During the study, the patients used on average 98.6 g of foam and 164.3 g of gel (after 4 and 8 weeks, respectively). They used 236.4 g of foam and 193.1 g of gel over 12 weeks. 

The secondary endpoints of effectiveness were the percentage of patients achieving at least 75% of modified PASI (mPASI75) reduction and the time to treatment success. A significant advantage of the foam was demonstrated also for those parameters. The authors emphasized that the median time for achieving the standard index of improvement of mPASI75 was 4 weeks for the foam and 12 weeks for the gel. 

A phase III clinical trial also assessed the influence of using foam and gel formulations on patients’ quality of life [27]. The following scales were used in the assessment: Health-Related Quality of Life (HRQoL), including the Dermatology Life Quality Index (DLQI), the EuroQoL-5D-5L-PSO (EQ-5D), and the Psoriasis QoL (PQoL-12). Moreover, the researchers evaluated such variables as pruritus, sleep deprivation triggered by pruritus, and the influence of the disease on the working life. The study included 463 patients with plaque psoriasis with BSA of 2 to 30%. In this group, 185 patients applied a foam, 188 used a gel formulation, 47 used a foam vehicle without active substances, and 43 used a gel vehicle. DLQI 0 or 1 were obtained by considerably more patients using the foam rather than the gel at week 4 (45.7% vs 32.4%, respectively; *p* = 0.013) and at week 12 (60.5% vs 44.1%, respectively; *p* = 0.003). The foam was also more effective as regards other parameters concerning the quality of life, including EQ-5D (0.09 vs 0.03; *p* < 0.001) and PQoL-12 (−2.23 vs −2.07; *p* = 0.029), and in terms of the influence on pruritus, pruritus-related sleep deprivation, and work impairment. 

Another phase III clinical trial [28] evaluated the preferences of patients concerning the vehicle. The authors compared a foam containing calcipotriol with a gel containing the same active substance. “The previous treatment” was the reference point. It was a prospective multicenter study (NCT02310646). The foam was used once daily for 7 days and then was substituted by the gel or the opposite. The study included 213 patients. For some parameters, the patients claimed they preferred the foam, e.g., because of immediate relief, the soothing quality of the preparation, or the feeling of alleviating the condition. As regards the gel, the patients indicated its easy application or easy spreading. 

## 6. Special Locations

The efficacy of gel, ointment, and foam formulations was not extensively studied in special locations which are difficult to treat. Some studies were conducted on the efficacy of calcipotriol/betamethasone in nail psoriasis. 

Rigopoulos et al. [29] treated 22 patients with nail psoriasis (114 nails) with an ointment once daily for 12 weeks. An average improvement of 72% was observed in the nail psoriasis severity index (NAPSI). Saki et al. [30] investigated 16 patients with nail psoriasis treated with an ointment formulation for 6 months and observed a mean improvement of 55.5% in the NAPSI. 

In a study performed by Gregoriou et al. [31], a calcipotriol/betamethasone foam was applied once daily on the proximal nail fold and hyponychium. The mean total NAPSI was reduced by 44%. Case reports also indicated the possible efficacy of a gel formulation monotherapy in nail psoriasis [32].

There were no head-to-head studies, but indirect data may indicate that the ointment formulation is more effective in nail psoriasis compared to foam.

The effect of a calcipotriol/betamethasone gel and foam in scalp psoriasis was extensively studied [33,34,35,36,37,38]. No head-to-head studies are available to evaluate the relative efficacy or safety of different formulations for scalp psoriasis. A 2017 Cochrane review of topical treatment options in scalp psoriasis indicated the presence of overall moderate-quality evidence confirming that the two-compound combination was only of small benefit over the formulation containing only the corticosteroid and that both therapies were similarly safe in short-term therapy. It was also concluded that there was overall moderate-quality evidence confirming that the two-compound combination was more effective and safer than vitamin D alone in short-term therapy [39]. Differences in the methodology of various short-term studies (4–8 weeks) performed in adult patients with scalp psoriasis limit the possibility of comparing the efficacy of these two formulations. The ointment formulation was not studied in isolated scalp psoriasis in clinical trials. A small case series indicated the possibility of significant efficacy [40]. However, difficulty in washing the ointment out of the hair may be a limiting factor [40].

A long-term study of a calcipotriol/betamethasone gel in adult patients performed by Saraceno et al. [36] indicated that maintenance therapy with twice-weekly applications versus on-demand treatment was more effective and was associated with a lower rate of relapse. The treatment was considered cosmetically acceptable by 79% of the patients [23].

Scalp psoriasis in adolescents was not extensively studied. An analysis of data from phase 2 studies was performed to evaluate the efficacy of a foam formulation in scalp psoriasis in adults and adolescents from the age of 12 years. An improvement was observed in PGA classification at week 4 in 73.6% of the adolescents and it was higher compared to that reached in the adults [38]. There are no studies focusing on the possible application of betamethasone/calcipotriol in patients with intertriginous or genital psoriasis. However, expert opinions indicate that a short course of treatment is likely to have a good efficacy and safety profile [41]. The comparison of the pharmaceutic properties may indicate a preference for foam over ointment or gel. 

## 7. Adverse Effects

The safety profile of foams was compared to that of ointments by Koo et al. [23] and to that of gels by Paul et al. [26]. The available safety data related to foams have recently been collected and analyzed by Amat-Samaranch and Puig [42]. The data showed that that the safety profiles of foams, ointments, and gels were comparable, with adverse effects observed in more than 1% of patients being application site pruritus (5.8%), skin atrophy (1.9%), folliculitis (1.9%), skin burning sensation (1.4%), skin depigmentation (1.4%), and erythema (1.0%). 

## 8. Calcipotriol/Betamethasone Foam as a Formulation Bridging the Gap between Topical and Systemic Treatment in Psoriasis 

A study published in December 2020 evaluating foam containing calcipotriol with betamethasone indicated the latter as a drug that might influence the costs of the biologic treatment of patients with the moderate-to-severe disease [43]. The authors indicated that biologic treatment was effective as a monotherapy in numerous patients suffering from psoriasis, while in some patients a change in treatment was necessary. The study aimed to analyze possible cost savings resulting from the use of a foam formulation combined with a systemic biologic treatment compared to a monotherapy treatment. The study included 30 patients. It was a 16-week, open-label, single-arm study of adjunctive therapy with a foam containing calcipotriol and betamethasone in patients who had been treated with etanercept or adalimumab for more than 24 weeks, without obtaining a satisfactory treatment response. The analysis involved the assessment of the affected body surface area (BSA), a general evaluation of disease severity performed by a doctor (PGA), BSA × PGA, NPF Treat to Target, and the probability of changing a biologic drug by the attending physician. Simultaneously, the authors analyzed the cost of treatment. Notably, the abundance of results obtained in the study showed that the probability of switching the biologic drug into another (potentially more expensive) decreased from 90.0% at baseline to 7.1% after 16 weeks of research which included 4 weeks of treatment with the topical drug applied once daily, followed by 12 weeks of maintenance/proactive therapy. The National Psoriasis FoundationTreat to Target status was achieved by over 75% of the patients by 4 weeks. The pharmacoeconomic assessment revealed that the adjuvant use of foam was more cost-effective compared to switching biologic drugs. In conclusion, the authors indicated that adjuvant therapy with calcipotriol and betamethasone dipropionate in a foam formulation may result not only in considerable clinical benefits but also in potential cost savings. 

A recent meta-analysis was performed to compare literature data referring to the effectiveness of a treatment with a foam containing calcipotriol and betamethasone compared to that of classic methods for the systemic treatment of psoriasis [44]. The time to therapeutic effect for each of the analyzed drugs was the reference point. Patients treated with a foam containing calcipotriol and betamethasone were characterized by a significantly better response assessed with the PASI75 scale compared to patients administered apremilast, methotrexate, and acitretin, and by a similar response compared to patients administered fumaric acid esters. Despite numerous methodological limitations of such analysis of literature data, the presented material reflects the questions frequently asked by dermatologists nowadays.

A similar presumption was the basis of a publication which analyzed the treatment costs of a foam containing calcipotriol and betamethasone and of systemic treatment [45]. The authors compared short-term costs and treatment effectiveness. The analysis comprised methotrexate, acitretin, fumaric acid esters, and apremilast. The authors assumed the perspective of the payer, which included the drug, medical appointments, and treatment monitoring as treatment costs. The lowest cost per responder (CPR) was generated by foam in all countries. It was calculated as the standard time necessary for achieving clinical response. The cost of treatment with foam was 190–359 Euros lower than that with methotrexate, apremilast, and acitretin. On the basis of the data in this publication, it may be concluded that in cases in which topical treatment is possible and clinically indicated, a foam with calcipotriol and betamethasone may constitute an attractive alternative for the short-term treatment of patients with psoriasis on the border of clinical indications for systemic treatment.

Bagel et al. [46] investigated whether patients who did not respond to systemic treatment with apremilast as monotherapy might be successfully treated for psoriasis (treatment effectiveness defined as PASI75) if a foam containing calcipotriol and betamethasone was introduced. The authors demonstrated that the majority of patients with partial response to treatment at week 8 might achieve PASI75 at week 12 with the combination therapy in topical foam and maintain PASI75 until week 16 with a systemic drug as monotherapy. It may indicate that even a short-term addition of foam containing calcipotriol to systemic treatment leads to an improvement and the maintenance of good response to the same drug, without the necessity of changing drugs in systemic treatment (Table 1). 

## 9. Proactive Maintenance Treatment

The chronic course of psoriasis and the tendency towards rapid relapses after topical treatment constitute a significant problem in the treatment of psoriasis. Topical corticoid treatments are approved for no more than 4–8 weeks, leaving the patient and physician with limited possibilities for treatment continuation. According to the registered posology regimen, the possibility of using a foam with calcipotriol and betamethasone as a proactive treatment for up to 52 weeks opens a new avenue for the long-term therapy of psoriasis. 

A phase III clinical trial (NCT02899962) described by Lebwohl et al. [49] and Stein Gold et al. [50] in 2020 collected positive results. It involved the assessment of the safety and effectiveness of a calcipotriol and betamethasone foam used twice weekly for 52 weeks as a proactive maintenance therapy aiming to prevent a relapse or achieve the longest possible clinical remission time. This idea is based on the long-term application of the foam to healthy-appearing areas following the resolution of skin lesions. 

In this context, it is worth emphasizing that the clinical regression of lesions is not equivalent to the resolution of the inflammatory process in the skin. A study conducted with the use of noninvasive skin imaging techniques indicated that an inflammation persisted in the skin despite achieving the apparent regression of skin lesions [51]. It was also demonstrated that the post-treatment dermoscopic picture of skin lesions allowed the determination of prognosis regarding the recurrence of cutaneous lesions in psoriasis [52]. The subclinical presence of an ongoing inflammatory process in the skin of patients with psoriasis explains the tendency towards rapid recurrences of psoriatic skin lesions in a significant proportion of patients treated with conventional 4–8 week courses of calcipotriol and betamethasone ointments or gels. This phenomenon identified in recent years has become one of the most important presumptions as regards the development of proactive psoriasis treatments which would offer a chance to prolong the remission time after topical treatment. 

The phase III clinical trial which was mentioned above [49] included 650 patients, with 521 being randomized to the proactive phase of maintenance therapy. The time to first relapse was the primary endpoint. A total of 251 randomized patients (46.1%) completed the study. The median of the time to first relapse was 56 days (in a group of patients undergoing proactive treatment) and 30 days (control group), which indicated that the time to first relapse was 87% longer in patients treated proactively. In total, the patients in the proactive treatment group had additional 41 days of remission over 52 weeks compared to the control group of patients in whom subsequent treatment cycles were introduced according to previous clinical practice, i.e., after a clinical relapse (*p* < 0.001). The number of relapses during 52 weeks was 3.1 (study group), which was 35% lower than in the control group (4.8). Moreover, the tendency towards rebounds was lower compared to that at baseline (PASI ≥ 125%). The safety profile was comparable for both therapeutic methods. 

In 2020, Kircik et al. [47] summarized available scientific evidence and literature data in a review article. Data analysis indicated that foams (but not other pharmaceutical formulations) containing calcipotriol and betamethasone have such a high anti-psoriatic effectiveness that, in some psoriasis patients, the decision to implement systemic treatment might be unnecessary. 

## 10. Conclusions

Calcipotriol/betamethasone foams show significantly higher efficacy compared to ointment and gel formulations in the treatment of plaque psoriasis. The higher clinical efficacy may be attributed to the supersaturation technique which was used for the production of the foam formulations. Gels and ointments have shown some benefits in the topical treatment of scalp and nail psoriasis, respectively. The available data indicate that the foam formulation may close the gap between topical and systemic therapy in plaque psoriasis, particularly when applied as a long-term proactive maintenance treatment.

## Figures and Tables

**Table 1:** Major differences between calcipotriol/betamethasone foam, ointment, and gel.

Differences between Calcipotriol/Betamethasone Gel, Foam, and Ointment
higher bioavailability of foam compared to ointment [19] higher clinical efficacy of foam compared to ointment and gel documented in clinical trials [22,23,24,26,27] higher efficacy of foam compared to gel as regards relieving pruritus [27] higher efficacy of foam compared to gel in relieving pruritus-related sleep disorders [27] higher efficacy of foam compared to gel as regards the influence on the quality of life [27] gel or foam are preferred by patients for their different practical qualities (e.g., gel for “easy application” and foam for “immediate relief”) [28] lower number of medical appointments with foam compared to ointment [25] lower probability of developing indications for systemic treatment or for switching to a different systemic treatment with foam in comparison with ointment [25,47], maintenance therapy markedly reduces the number of relapses (approved for foam, not for ointment or gel) ointment appears to be more effective compared to foam in nail psoriasis (no head-to-head data) [30,31,48] the efficacy of foam and gel in scalp psoriasis was studied with inequivalent methodologies; gel is cosmetically acceptable by 79% of patients [36,38]
